# The ROS/NF-κB/NR4A2 pathway is involved in H_2_O_2_ induced apoptosis of resident cardiac stem cells via autophagy

**DOI:** 10.18632/oncotarget.20747

**Published:** 2017-09-08

**Authors:** Xingxing Shi, Wenjing Li, Honghong Liu, Deling Yin, Jing Zhao

**Affiliations:** ^1^ Shandong Provincial Key Laboratory of Animal Cells and Developmental Biology, School of Life Science, Shandong University, Jinan 250100, China; ^2^ Department of Pharmacology, School of Pharmaceutical Sciences, Central South University, Changsha 410078, China; ^3^ Department of Internal Medicine, College of Medicine, East Tennessee State University, Johnson City, TN 37614, USA

**Keywords:** NR4A2, cardiac stem cells, autophagy, apoptosis, ROS

## Abstract

Cardiac stem cells (CSCs)-based therapy provides a promising avenue for the management of ischemic heart diseases. However, engrafted CSCs are subjected to acute cell apoptosis in the ischemic microenvironment. Here, stem cell antigen 1 positive (Sca-1^+^) CSCs proved to own therapy potential were cultured and treated with H_2_O_2_ to mimic the ischemia situation. As autophagy inhibitor, 3-methyladenine (3MA), inhibited H_2_O_2_-induced CSCs apoptosis, thus we demonstrated that H_2_O_2_ induced autophagy-dependent apoptosis in CSCs, and continued to find key proteins responsible for the crosstalk between autophagy and apoptosis. Nuclear Receptor Subfamily 4 Group A Member 2 (NR4A2), increased upon cardiomyocyte injury with unknown functions in CSCs, was increased by H_2_O_2_. NR4A2 siRNA attenuated H_2_O_2_ induced autophagy and apoptosis in CSCs, which suggested an important role of NR4A2 in CSCs survival in ischemia conditions. Reactive oxygen species (ROS) and NF-κB (P65) subunit were both increased by H_2_O_2_. Either the ROS scavenger, N-acetyl-l-cysteine (NAC) or NF-κB signaling inhibitor, bay11-7082 could attenuate H_2_O_2_-induced autophagy and apoptosis in CSCs, which suggested they were involved in this process. Furthermore, NAC inhibited NF-κB activities, while bay11-7082 inhibited NR4A2 expression, which revealed a ROS/NF-κB/NR4A2 pathway responsible for H_2_O_2_-induced autophagy and apoptosis in CSCs. Our study supports a new clue enhancing the survival rate of CSCs in the infarcted myocardium for cell therapy in ischemic cardiomyopathy.

## INTRODUCTION

Ischemic heart disease remains one of the leading causes of death worldwide [1]. Cardiac stem cells (CSCs) showed great therapeutic potential in repairing and regenerating damaged hearts [2–4]. Among various types of stem cells, Sca-1^+^ CSCs accounted for the largest proportion and appeared to be particularly promising in repairing cardiac damage [5]. However, the low survival of engrafted stem cells caused by acute cell apoptosis still remains a major challenge for stem cell therapy [6]. To this end, it is pivotal to search for new targets to inhibit CSC apoptosis in oxidative stress situation in order to foster the success of stem cell-based therapy.

Autophagy is a cell survival process breaking down and reusing cytoplasm components [7]. Autophagy may cooperate with the apoptotic machinery to regulate cell death under certain conditions [8]. Due to the relatively long life of stem cells, autophagy should be indispensable for the quality control and maintenance of cellular homeostasis [7]. In spite of the rich knowledge available for somatic cells, the precise role of autophagy in the maintenance and function in stem cells is only beginning to be understood [9, 10]. It reported that autophagy participated in the therapeutic efficacy of apelin on transplanted mesenchymal stem cells in hindlimb ischemic mice, which revealed the potential importance of autophagy on the survival of transplanted stem cells [11]. Recent evidence also suggested that autophagy might serve as a therapeutic target in the management of ischemia/reperfusion injury [12, 13]. To target autophagy is a new method inhibiting apoptosis, but proteins modulate both autophagy and apoptosis were less reported.

NR4A1 and NR4A2 are closely related nuclear orphan receptors with increased expression upon cardiomyocyte injury. In previous studies, NR4A1 has been proofed to play an important function in the induction of both autophagy and apoptosis in cardiomyocytes [14, 15], but the role of NR4A2 in the heart is not clear. It is reported that NR4A2 was involved in neuronal degeneration [16], as many of the proteins regulating neuronal degeneration also participated in autophagy [17], we deduced that NR4A2 might be important in the connection between autophagy and apoptosis. Till now, NR4A2 was reported to regulate differentiation, proliferation and migration of various stem cells [18–20], but its roles on autophagy and apoptosis in CSCs remain unclear.

Reactive oxygen species (ROS) -activated NF-κB signaling was responsible for H_2_O_2_ induced apoptosis in various cell lines [21, 22]. As they also participated in autophagy [23, 24], and regulated NR4A2 expression in indicated conditions [25, 26], we deduced that they were the upstream factors regulating NR4A2.

Here we reported that H_2_O_2_ induced autophagy-dependent apoptosis through the ROS/NF-κB/NR4A2 signaling pathway. Suppression of autophagy could down-regulate apoptosis in the CSCs under oxidative stress.

## RESULTS

### H_2_O_2_ induced apoptosis of resident cardiac stem cells

**Figure 1:**
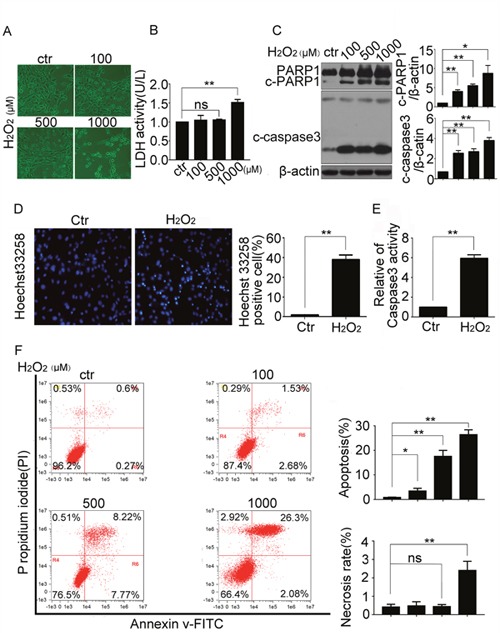
H_2_O_2_ induced apoptosis of resident cardiac stem cells CSCs were treated with indicated concentrations of H_2_O_2_ for 5 h. **(A)** The morphologic changes were observed with a phase contrast microscope (Scale bar=100 μm). **(B)** The LDH activity were detected by the LDH detection kit. **(C)** Western blot showed the protein levels of apoptosis-related proteins caspase3 and poly (ADP-ribose) polymerase 1 (PARP1). **(D** and **E)** CSCs were treated with H_2_O_2_ (500 μM) for 5 h, then the cells were stained with Hoechst 33258 to show the DNA fragmentation and condensation of CSCs (Scale bar=20 μm) (D), and caspase3 activity was analyzed (E). **(F)** The FCM showed the necrosis and apoptosis ratio of CSCs treated with indicated concentrations of H_2_O_2._ ctr, control. *P < 0.05; **P < 0.01; n=3.

To establish an apoptosis model to mimic the *in vivo* situation of infarction, the oxidase stress generating agent, H_2_O_2_, was used in this study [14, 15]. CSCs were treated with indicated concentrations of H_2_O_2_ (100, 500, and 1000 μM) for 5 h. The morphology changes showed that H_2_O_2_ induced apoptosis-like cells (Figure 1A), but LDH detection showed that H_2_O_2_ promoted necrosis at more than 500 μM (Figure 1B). To detect apoptosis, the cleavage of apoptosis-related proteins caspase 3 and poly (ADP-ribose) polymerase 1 (PARP1), nuclear fragment and caspase 3 activity were detected by western blot (Figure 1C), Hoechst 33258 staining (Figure 1D) and caspase 3 activity detection kit (Figure 1E) respectively. The necrosis and apoptosis ratio of CSCs treated with indicated concentrations of H_2_O_2_ were detection by FCM (Figure 1F). All the data revealed that H_2_O_2_ less than 500 μM induced apoptosis but not necrosis of resident CSCs. Therefore, we chosen a concentration of 500 μM in the followed mechanism study.

### H_2_O_2_ induced autophagy of resident cardiac stem cells

**Figure 2:**
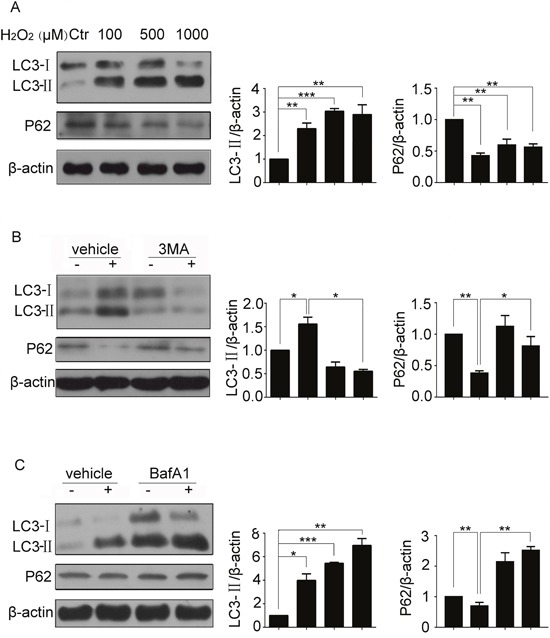
H_2_O_2_ induced autophagy of resident CSCs **(A)** CSCs were treated with indicated concentrations of H_2_O_2_ for 5 h, changes of LC3-II were observed by western blot. **(B** and **C)** CSCs were pretreated with 3MA (5 mM) or Baf A1 (50 nM) for 30 min followed by H_2_O_2_ treatment for 5 h. The levels of LC3-II and P62 were detected by western blot. ctr, control. *P < 0.05; **P < 0.01; n=3.

Autophagy could regulate cell apoptosis, to determine whether H_2_O_2_ induced autophagy, we treated CSCs with H_2_O_2_ at various concentrations. The results showed that LC3-II levels increased by H_2_O_2_ (Figure 2A), the increasing of LC3-II might be caused by the autophagic initiation promotion or autophagic flux blocking. To distinguish the reasons responsible for the accumulation of LC3-II, we measured protein levels of P62, a selective substrate of autophagy. As Figure 2A shows, P62 levels decreased by H_2_O_2_. Then, 3MA, blocker for autophagic initiation, was added to CSCs. As a result, LC3-II levels were markedly attenuated by 3MA, and P62 levels were reversed by 3MA, which suggested that H_2_O_2_ promoted the initiation of autophagy (Figure 2B). Baf A1 could inhibit fusion of lysosomes and autophagosomes, and used as a blocker for autophagic flux. When Baf A1 was added, LC3-II protein levels were increased furtherly, P62 levels were reversed by Baf A1, which suggested that H_2_O_2_ did not disrupt the autophagic flux (Figure 2C).

### H_2_O_2_-induced apoptosis in CSCs was regulated by autophagy

**Figure 3:**
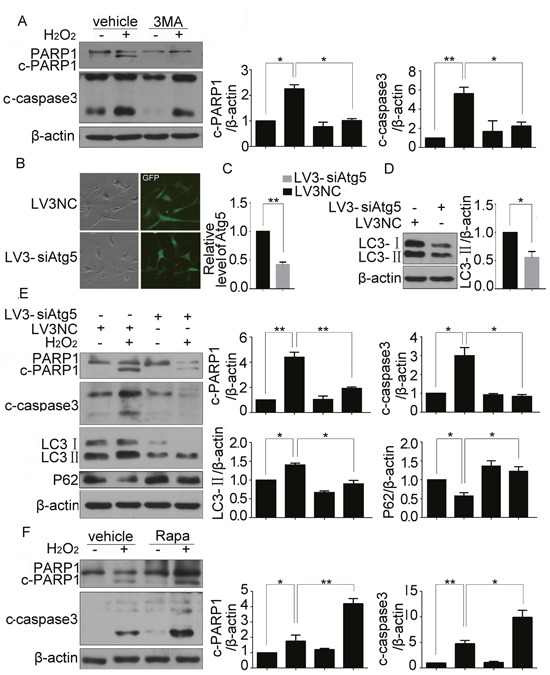
H_2_O_2_-induced apoptosis in CSCs was regulated by autophagy CSCs were pretreated with 3MA for 30 min before incubating with H_2_O_2_ for 5 h. **(A)** Western blot showed protein levels of cleaved caspase3 and PARP1. **(B)** The fluorescent photo showed the transfection ratio of Atg5 siRNA. **(C)** qPCR showed the efficiency of Atg5 knockdown. **(D)** Western blot showed the changes of LC3-II in cells with Atg5 knockdown. **(E)** CSCs were transfected with Atg5 siRNA for 43 h followed by H_2_O_2_ treatment for another 5 h. cleaved caspase3, PARP1, LC3-II and P62 were detected by western blot. **(F)** CSCs were pretreated with Rapamycin for 30 min before incubating with H_2_O_2_ for 5 h. Western blot showed protein levels of cleaved caspase3 and PARP1. *P < 0.05; **P < 0.01; n=3.

To investigate the functional of autophagy in H_2_O_2_-caused CSC apoptosis, we inhibited the autophagic response in H_2_O_2_ treated CSCs with 3MA, and found that 3MA pretreatment reversed H_2_O_2_ induced cleavage of caspase 3 and PARP1 (Figure 3A). Atg5 is an E3 ubiquitin ligase which is necessary for autophagy due to its role in autophagosome elongation, lentiviral-mediated stable ablation of Atg5 was performed in CSCs with its siRNA. When the lentiviral entered into CSCs (Figure 3B), the siRNA effectively silenced the expression of endogenous Atg5 in CSCs (Figure 3C), at the same time, LC3-II protein levels was markedly attenuated because autophagy was inhibited (Figure 3D). Results of Figure 3E showed that Atg5 knockdown attenuated the cleavage of caspase3 and PARP1, and reversed changes of LC3-II and P62 induced by H_2_O_2_. In contrast, Rapamycin, a well-known autophagy inducer through inhibiting mTORC1, aggravated H_2_O_2_ induced cleavage of caspase 3 and PARP1 (Figure 3F). The results confirmed thatH_2_O_2_-induced apoptosis in CSCs was regulated by autophagy.

### NR4A2 was involved in H_2_O_2_-induced autophagy and apoptosis in CSCs

**Figure 4:**
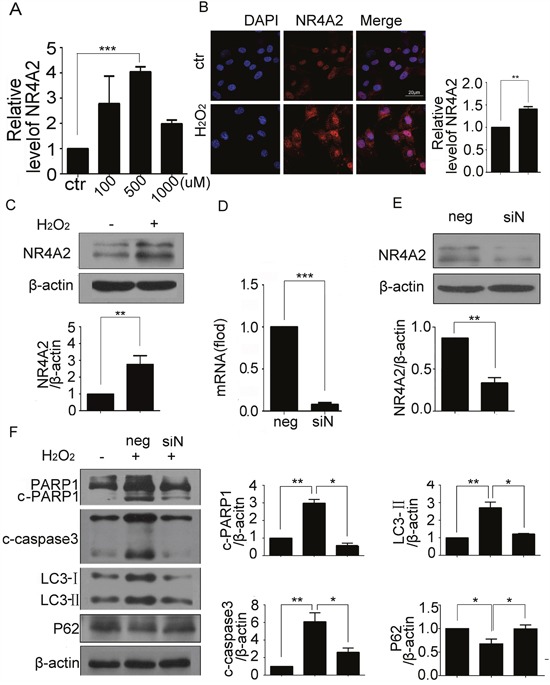
NR4A2 knockdown inhibited H_2_O_2_-induced autophagy and apoptosis in CSCs CSCs were challenged with indicated concentrations of H_2_O_2_ for 5 h. **(A)** Transcription of NR4A2 was observed by qPCR. **(B)** Representative immunofluorescent images showed the expression of NR4A2 in CSCs treated with H_2_O_2_. Scale bar=20 μm. **(C)** NR4A2 protein levels were determined by western blot. **(D** and **E)** CSCs were transfected with NR4A2 siRNA for 48 h, then the mRNA and protein were extracted for qPCR detection (D) and western blot (E). **(F)** CSCs were transfected with NR4A2 siRNA for 43 h followed by H_2_O_2_ treatment for another 5 h. cleavage of caspase 3 and PARP1, LC3-II and P62 were detected by western blot. neg, negative control, siN, siNR4A2, ctr, control. *P < 0.05; **P < 0.01; n=3.

**Figure 5:**
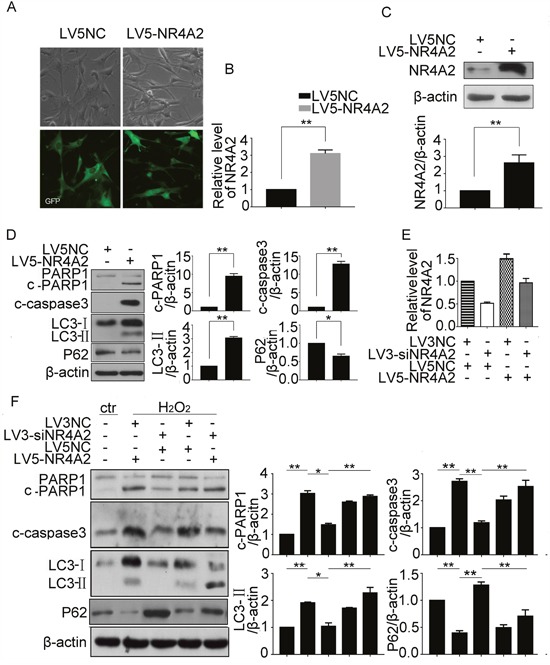
NR4A2 participated in H_2_O_2_-induced autophagy and apoptosis in CSCs CSCs were treated with LV5-NR4A2 for 48 h. **(A)** The fluorescent images showed the transfection efficiency. **(B** and **C)** NR4A2 changes in mRNA and protein were detected by qPCR (B) and western blot (C). **(D)** cleavage of caspase 3 and PARP1, LC3-II and P62 were detected by western blot. **(E)** CSCs with NR4A2 knockdown were transfected with NR4A2 overexpression plasmid or the negative control, then NR4A2 changes in mRNA and protein were detected by qPCR. **(F)** cleavage of caspase 3 and PARP1, and LC3-II and P62 in H_2_O_2_-treated CSCs were detected by western blot. *P < 0.05; **P < 0.01; n=3.

We then sought to character the mediators implicated in autophagy-dependent apoptosis in CSCs. To evaluate the importance of NR4A2, the mRNA and protein expression were evaluated by qPCR, immunofluorescence, and western blot respectively. All the results showed that H_2_O_2_ could enhance the expression of NR4A2 at mRNA and protein levels (Figure 4A, 4B, 4C), and the immunofluorescence showed that H_2_O_2_ promoted cytoplasmic expression of NR4A2 compared with control (Figure 4B). Then we used NR4A2 siRNA to knockdown its expression, qPCR and western blot results showed that siRNA at 80 nM could down regulate NR4A2 expression significantly (Figure 4D, 4E). Furthermore, NR4A2 siRNA reversed the cleavage of caspase 3 and PARP1, LC3-II increasing and P62 decreasing induced by H_2_O_2_ (Figure 4F). To be more confident that NR4A2 participates in H_2_O_2_-induced autophagy and apoptosis, we use LV5-NR4A2 overexpression plasmid, the fluorescent images showed the transfection efficiency (Figure 5A), qPCR and western blot results showed that LV5-NR4A2 could upregulate NR4A2 expression significantly (Figure 5B, 5C). As a result, overexpression of NR4A2 induced cleavage of caspase3 and PARP1, LC3-II increasing and P62 decreasing, the same as H_2_O_2_ induced (Figure 5D). Furthermore, CSCs with NR4A2 knockdown were transfected with NR4A2 overexpression plasmid or the negative control, qPCR and western blot demonstrate that NR4A2 overexpression rescued the effect induced by NR4A2 knockdown (Figure 5E, 5F), which verified that NR4A2 was involved in H_2_O_2_-induced autophagy and apoptosis in CSCs.

### NR4A2 mediated autophagy-dependent apoptosis in H_2_O_2_-induced autophagy and apoptosis in CSCs

**Figure 6:**
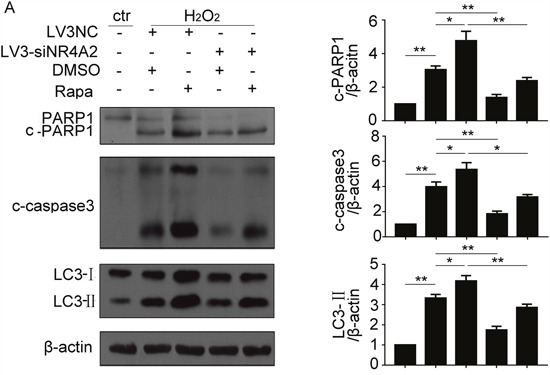
NR4A2 mediated autophagy-dependent apoptosis in H_2_O_2_-induced autophagy and apoptosis in CSCs **(A)** CSCs were transfected with LV3-NC or LV3-siNR4A2 for 48 h, then pretreated with Rapamycin for 30 min followed by H_2_O_2_ treatment for another 5 h. cleavage of caspase 3 and PARP1, and LC3-II were detected by western blot. *P < 0.05; **P < 0.01; n=3.

To clarify the relationship between NR4A2-mediated autophagy and apoptosis, lentiviral-mediated stable ablation of NR4A2 was performed in CSCs with its siRNA combined with two tools of autophagy. As shown in Figure 6A, H_2_O_2_ stimulated cleavage of caspase 3 and PARP1, and LC3-II levels was attenuated by NR4A2 knockdown (Figure 6A). Furthermore, Rapamycin increased their changes. Thus, NR4A2 mediated autophagy-dependent apoptosis in H_2_O_2_-induced autophagy and apoptosis in CSCs.

### NR4A2 was regulated by NF-κB directly in H_2_O_2_-induced autophagy and apoptosis in CSCs

**Figure 7:**
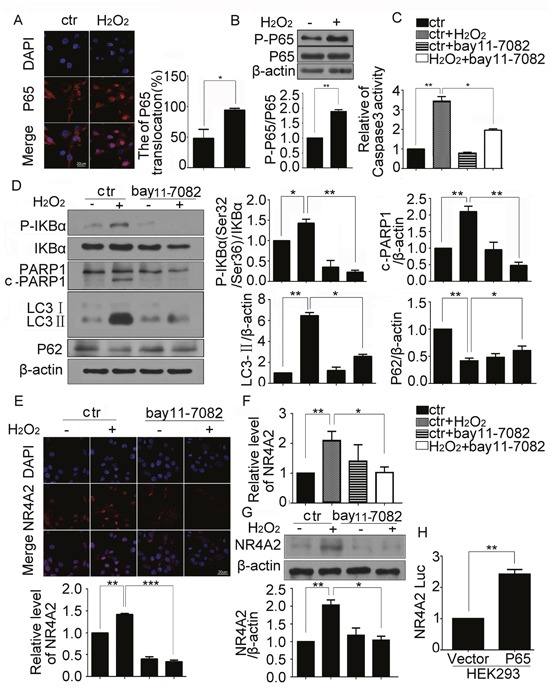
NR4A2 was regulated by NF-κB directly in H_2_O_2_-induced autophagy and apoptosis of CSCs CSCs were challenged with 500 μM H_2_O_2_ for 5 h. **(A)** Representative immunofluorescent images showed the translocation of P65 in CSCs. **(B)** Change of phosphorylated P65 were detected by western blot. **(C)** The caspase3 activity was detected by Caspase3 Colorimetric Assay kit. **(D)** CSCs were pretreated with NF-κB inhibitor bay11-7082 (10 μM) for 30 min, then treated with H_2_O_2_ for 5 h, western blot showed the changes of phosphorylated IκBα, cleaved PARP1, LC3-II and P62 in CSCs. **(E–G)** The changes of NR4A2 in CSCs treated with bay11-7082 combined with H_2_O_2_ were detected by immunofluorescent assay (E), the qPCR (F), western blot (G) and respectively. **(H)** Luciferase reporter plasmid containing the NR4A2 promoter was co-transfected with Flag-P65-AMP overexpression plasmid in HEK293 cells for 24 h followed by dual luciferase activity assay. ctr, control. *P < 0.05; **P < 0.01; n=3.

Transcription factor NF-κB was involved in the apoptosis of various cells treated with H_2_O_2_. The activation of NF-κB was associated with its nucleus translocation and phosphorylation. To determine whether NF-κB participated in H_2_O_2_ induced autophagy and apoptosis in CSCs, nucleus translocation and phosphorylation of its P65 subunit were detected respectively. The immunofluorescence results showed that H_2_O_2_ induced the translocation of P65 to the nucleus (Figure 7A), and western blot results showed that H_2_O_2_ increased P65 phosphorylation in CSCs (Figure 7B), which suggested that NF-κB was activated in H_2_O_2_-treated CSCs. bay11-7082, the specific inhibitor of NF-κB, impaired NF-κB activity through inhibiting IκB phosphorylation. We examined caspase 3 activation and got a coincident result (Figure 7C). Western blot results showed decreased phosphorylation of IκB, cleaved PARP1 and LC3-II levels in CSCs pretreated with bay11-7082 (Figure 7D). Our data confirmed that NF-κB was involved in H_2_O_2_-induced autophagy and apoptosis in CSCs. To evaluate the regulation f P65 to NR4A2, the expression of NR4A2 was evaluated by qPCR, immunofluorescence, and western blot respectively. All the results showed that bay11-7082 reduced expression of NR4A2 at mRNA and protein levels (Figure 7E, 7F, 7G). There is a NF-κB binding site located within the NR4A2 promoter [26], in order to further confirm that NF-κB regulate NR4A2 expression directly, we cloned mus-NR4A2 promoter region into luciferase reporter vector pGL3-basic. Luciferase reporter plasmid was co-transfected with Flag-P65-AMP overexpression plasmid in HEK293 cells, the luciferase reporter results showed that NR4A2 Luc was increased in cells transfected with Flag-P65-AMP overexpression plasmid compared with.

### ROS was the upstream of NF-κB/NR4A2 in H_2_O_2_-induced autophagy and apoptosis in CSCs

**Figure 8:**
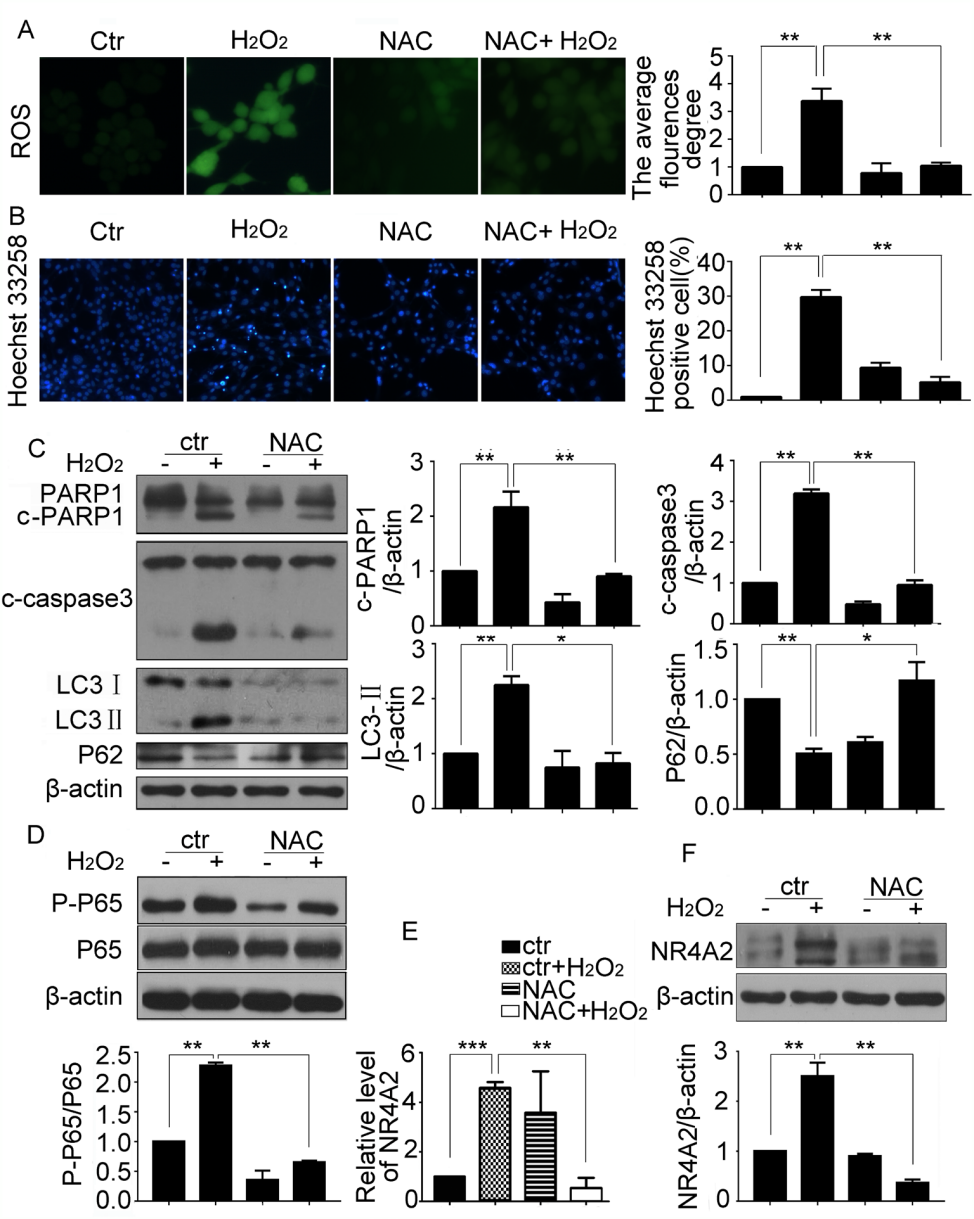
ROS was the upstream of NF-κB/NR4A2 in H_2_O_2_-induced autophagy and apoptosis in CSCs CSCs were treated with H_2_O_2_ (500 μM) in the absence or presence of the antioxidant NAC (0.5 mM) for 5 h. **(A)** Representative images (× 200) were presented to show ROS levels. **(B)** Hoechst 33258 staining showed the DNA fragmentation and condensation. Scale bar=20 μM. **(C)** Western blot results showed changes of cleavage of caspase 3 and PARP1, LC3-II and P62. **(D)** Western blot showed the changes of phosphorylated P65. **(E** and **F)** Changes of NR4A2 levels were detected by qPCR (E) and western blot (F). ctr, control. *P < 0.05; **P < 0.01; n=3.

ROS increased in the oxidase stress conditions, to investigate whether ROS plays a role in H_2_O_2_-induced autophagy and apoptosis in CSCs, the generation of ROS was measured by a fluorescence microscope. The results showed that H_2_O_2_ increased ROS levels in CSCs, and antioxidant NAC attenuated its production (Figure 8A). As NAC inhibited DNA fragmentation (Figure 8B), reversed H_2_O_2_ induced cleavage of caspase3 and PARP1, and LC3-II increasing and P62 decreasing (Figure 8C), we concluded that H_2_O_2_-induced ROS contributed to autophagy and apoptosis of CSCs. Previous reports showed that ROS could activate NF-κB [23], we deduced that ROS/NF-κB/NR4A2 pathway existed in H_2_O_2_-induced CSC autophagy and apoptosis. To confirm our hypotheses, we blocked ROS with its inhibitor NAC. qPCR and western blot displayed that NAC reversed H_2_O_2_ stimulated phosphorylation of P65 (Figure 8D), and NR4A2 expression (Figure 8E, 8F). The results confirmed that ROS/NF-κB/NR4A2 pathway existed in H_2_O_2_-induced CSC autophagy and apoptosis.

## DISCUSSION

The low survival rate of transplanted CSCs caused by apoptosis hampers the efficiency of cell therapy in ischemic heart diseases. We established an *in vitro* oxidative stress model with H_2_O_2_ to mimic the microenvironment of infarcted myocardium. Given that 500 μM H_2_O_2_ induced the highest apoptosis and relatively low necrosis in Sca-1^+^ CSCs, we chose 500 μM H_2_O_2_ to study the mechanisms.

Accumulating evidence has consolidated for a major role of autophagy in a variety of physiological processes, including inflammation, oxidative stress, autophagic cell death and immune responses [27], but its roles on the survival of stem cells were less reported. Here, we reported that the apoptosis of CSCs were regulated by autophagy, so the tools inhibiting autophagy might be used together with CSCs to enhance the survival of CSCs.

The crosstalk between autophagy and apoptosis was existed, but the proteins responsible the crosstalk were less reported. It is reported that NR4A2 acts as necrosis promoter in HeLa cells during oxidative stress [28], but the roles of it in CSCs is unknown. We showed that NR4A2-mediated oxidative stress-induced autophagy results in less CSCs apoptosis and increased cell protection, so NR4A2 is a new protein mediated the crosstalk between autophagy and apoptosis, and might act as a target to enhance CSCs survival. Although NR4A2 is a well-known nuclear-localized transcription factor, it translocated from the nucleus into the cytosol when inducing necrosis in H_2_O_2_-treated HeLa cells. In this study, we also found the cytosol translocation of NR4A2 in inducing apoptosis (Figure 4C), but the mechanism needs further study.

Although the previous reports demonstrated that p38 could regulated the transcriptional activity and translocation of NR4A2, but more pathways regulating NR4A2 was not clear. In this study, we elucidated the roles of ROS/NF-κB pathway-dependent expression of NR4A2 in oxidative stress-induced apoptosis. Our data suggested that ROS/NF-κB pathway-dependent expression of NR4A2 is a crucial regulation to autophagy-dependent apoptosis.

It is generally believed that oxidative stress is a strong proautophagic stimulus. However, some evidence coming from neurobiology as well as from other fields indicate an inhibitory role of ROS on the autophagic machinery. Our data confirmed that ROS promoted autophagy and apoptosis in H_2_O_2_-treated CSCs.

**Figure 9:**
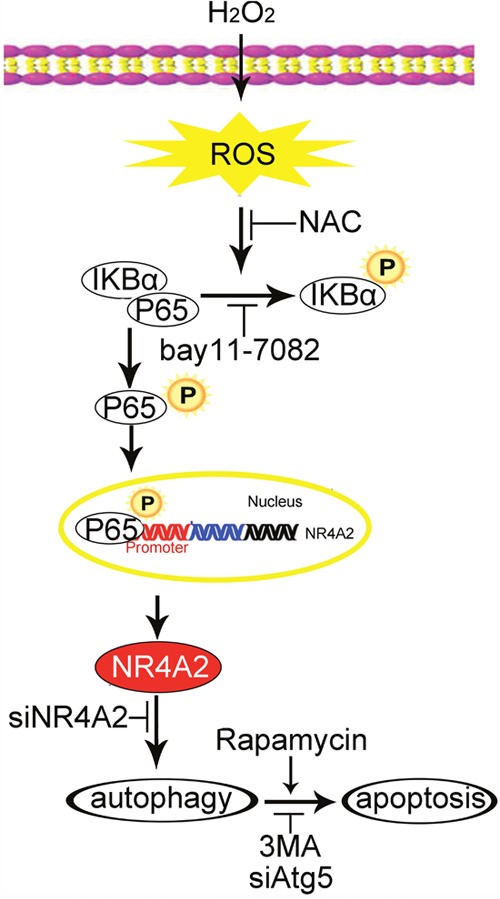
The graphic image shows that H_2_O_2_ induced autophagy-dependent apoptosis of CSCs ROS/P65/NR4A2 pathway was involved in this process. NAC, ROS scavenger; bay 11-7082, P65 inhibitor; 3MA, autophagy inhibitor; Rapamycin, autophagy inducer.

In summary, our data reveal that NR4A2 was involved in H_2_O_2_-induced apoptosis of resident cardiac stem cells through promoting autophagy. ROS and NF-κB were in the upstream of NR4A2 in H_2_O_2_-induced apoptosis and autophagy (Figure 9). Our data supports new clue for enhancing the survival of transplanted CSCs.

## MATERIALS AND METHODS

### Reagents

IMDM was from Invitrogen (Carlsbad, CA). siRNA of NR4A2 and negative control were from GenePharma (Shanghai, China). Lipofectamine 2000 was from Invitrogen (Carlsbad, CA). NR4A2 and β-actin antibodies were from Santa Cruz Biotechnology (Santa Cruz, CA, USA). Antibodies for caspase3, PARP1, p-P65, P65, p-IκBα, IκBα, LC3 were from Cell Signaling Technology (Beverly, MA, USA). 3-methyladenine (3MA) and N-acetyl cysteine (NAC) were from Sigma–Aldrich, bafilomycin A1 (Baf A1) was from Sangon Biotech (Shanghai, China). bay11-7082 was from Absin Inc. (Shanghai, China), antibody for Sca-1 and beads were all from BD Biosciences (San Jose, CA, USA). Caspase3 activity detection kit was from KeyGEN Biotech.

### Cell isolation, culture and treatment

Cardiac Sca-1^+^ cells were isolated from adult C57BL/6 mice by magnetic cell sorting with about 90% purity, and sub-cultured on 0.2% gelatin-coated dishes with IMDM supplemented with 10% fetal bovine serum (FBS, HyClone), as described previously [29]. During the experiment, CSCs were treated with medium deprived of FBS but supplement with H_2_O_2_.

### Western blot analysis

CSCs were lysed in RIPA lysis buffer on ice and PMSF was added as the protease inhibitor. Equal amount of proteins (20 μg) underwent 12% SDS-PAGE and then transferred to polyvinylidene difluoride (PVDF) membrane. The membrane was blocked in TBST containing 5% non-fat milk for 1 h. Membrane was incubated with primary antibody diluted in TBST (1:1000) at 4 °C overnight. After 3 washings in PBST, the PVDF membrane was incubated with appropriate horseradish peroxidase-conjugated secondary antibodies (1:5000) for 1 h at room temperature. The immunoreactive bands were developed with the ECL western blotting system. β-actin was used as loading control. The relative levels of proteins were analyzed with ImageJ software (National Institutes of Health, USA).

### Immunofluorescence staining

Immunofluorescence staining was done as reported [30]. Briefly, the Sca-1^+^ enriched cells were fixed with 4% paraformaldehyde for 15 min at room temperature and stained with anti-NR4A2 or anti-P65 antibodies, then Alexa Fluor 546-conjugated secondary antibody (Molecular Probes, Eugene, OR, USA). DAPI was used to stain the nucleus of cells. Laser scanning confocal microscopy (Leica, Wetzlar, Germany) was used for fluorescence detection. Images are representative of three independent experiments.

### Quantitative real-time PCR (qPCR)

Total RNA was extracted from CSCs using the TRIzol reagent (Invitrogen). Total RNA was extracted and reverse-transcribed into cDNA. qPCR involved the use of SYBR GreenER on the Bio-Rad PCR instrument. PCR reaction conditions followed the standard protocol. β-actin was used as an endogenous control. All qPCR reactions were performed in triplicate, and relative quantification involved the DDCt method (95% CI). Primer sequences for NR4A2, Forward Primer 5-GTGTTCAGGCGCAGTATGG-3, Reverse Primer 5-TGGCAGTAATTTCAGTGTTGGT-3, β-actin, Forward Primer 5’-AAGATCAAGATTGCTCCTC-3’ and Reverse Primer 5’-GGACTCATCGTACTCCTG-3’.

### LDH assay

When the cells cultured on 6-well cell culture plate reached sub-confluence, the cultures were changed with the IMDM deprived of serum but supplemented with indicated concentrations of H_2_O_2_. After treatment for 5 h, the LDH release was detected by LDH cytotoxicity assay kit (Cayman, Ann Arbor, MI, USA).

### Caspase3 activity detection

Cells were lysed in lysis buffer, then the supernatant of lysate was collected. After incubated with the reaction buffer and Caspase-3 Substrate for 4 h at 37°C, the supernatant were detected by ELX800 ( Bio-rad, USA).

### Hoechst 33258 staining

CSCs were grown in a 24-well plate and treated with H_2_O_2_ for 5 h or left untreated (control). CSCs were fixed with 4% paraformaldehyde and stained with 1 μg ml^−1^ Hoechst 33258 (Molecular Probes, Eugene, OR, USA) for 5 min. Cells were then washed twice with PBS and visualized with a Laser scanning confocal microscopy (Leica, Wetzlar, Germany).

### Intracellular ROS detection

Following H_2_O_2_ treatment, intracellular ROS was detected by fluorescence microscope using dichlorofluorescein diacetate (DCFH-DA) staining. Briefly, the CSCs were incubated with 0.5 mM DCFH-DA (Sigma-Aldrich) for 30 min at 37 C in the dark, then washed with serum-free medium for three times. The fluorescence was excited at the wavelength of 485 nm and the corresponding emission wavelength was 520 nm.

### Transfection of NR4A2 siRNA

CSCs were seeded in 60 mm cell culture dishes and cultured overnight. To knockdown the expression of NR4A2, cells at 40-60% confluence were transfected with NR4A2 siRNA using Lipofectamine 2000 reagent according to the manufacturer's instructions. At 43 h after transfection, CSCs were treated with medium deprived of FBS but supplement with H_2_O_2_ (500 μM) for 5 h. NR4A2 siRNA, 5’- CGATTTCTTAACTCCAGAGTT-3’ Negative control (NC), 5'-TTCTCCGAACGT GTCACGT-3'.

### Lentivirus infection

To stably knock down endogenous Atg5 and NR4A2 expression, we used lentivirus packing siRNA expression vector (pGLV3-GFP/Puro, GenePharma, Shanghai, China). Target cells were infected with lentivirus for 24–48 h according to the manufacturer's instruction. The RNAi oligonucleotides sequence used to knock down endogenous Atg5 and NR4A2 expression and its negative control were as follows: Atg5 siRNA, 5'-GCAGAACCATACTATTTGCTTCTC-3'; NR4A2 siRNA, 5’- CGATTTCTTAACTCCAGAGTT-3’; Negative control (NC), 5'-TTCTCCGAACGT GTCACGT-3'. To upregulate the expression of NR4A2, RNA overexpression vector (pGLV5-GFP/Puro, GenePharma, Shanghai, China) instead of siRNA was used.

### Cell apoptosis detection by flow cytometric (FCM) analysis

CSCs seeded in 10 cm-diameter dishes were treated with H_2_O_2_ at the indicated concentrations for 5 h, then collected and stained with Annexin V-FITC/PI apoptosis kit (BioLegend, California, USA) according to the manufacturer's instructions, and detected by flow cytometer (Image StreamX MarkII, Amnis, USA). The results were analyzed by IDEAS software (Amnis, USA).

### Luciferase assay analysis

We cloned mus-NR4A2 promoter region into luciferase reporter vector pGL3-basic, and prepared P65 overexpression vector (Flag-AMP, GenePharma, Shanghai, China). HEK293 Cells were co-transfected with 400 ng luciferase reporter vector pGL3-basic and Flag-P65-AMP for 24 h, then the luciferase assays were performed with a luciferase assay kit (Promega, Madison, WI).

### Statistical analysis

All data were presented as mean ± SEM. Student t test or oneway ANOVA analysis was performed using GraphPad Prism 6. P value <0.05 was considered to indicate a statistically significant different.

## Abbreviations

CSCs: Cardiac stem cells
Sca-1+: stem-cell antigen 1-positive
ROS: Reactive oxygen species
3MA: 3-methyladenine
NR4A2: Nuclear Receptor Subfamily 4 Group A Member 2
NAC: N-acetyl-l-cysteine
Baf A1: bafilomycin A1
PARP1: (ADP-ribose) polymerase 1
siRNA: silencing RNA
PMSF: Phenylmethylsulfonyl fluoride
LDH: Lactate dehydrogenase
Baf A1: bafilomycin A1
